# Whole genome sequencing of nine *Vibrio parahaemolyticus* strains encoding (*Pir*) toxin-like genes from shrimp cultures in northern Peru using Oxford Nanopore technology

**DOI:** 10.1128/mra.00511-23

**Published:** 2024-02-12

**Authors:** Stephanie Tapia-Chirinos, Luis Alberto Salcedo-Mejia, Luz Domínguez-Mendoza, Jeferson Nuñure-Ortega, Fernando Ramos-Espinoza, Muriel Gómez-Sánchez, Luis Tataje-Lavanda, Rodolfo Velazco-Peña

**Affiliations:** 1 Laboratorio de Sanidad Acuícola-Sede Tumbes, Organismo Nacional de Sanidad Acuícola (SANIPES), Lima, Peru; 2 Escuela Profesional de Medicina Humana, Universidad Privada San Juan Bautista, Lima, Peru; Montana State University, Bozeman, Montana, USA

**Keywords:** *Vibrio parahaemolyticus*, genetic diversity, shrimp cultures, PirAB genes, aquaculture health, pathogenic bacteria, antibiotic resistance, transposable elements, Peruvian farmed shrimp, disease control

## Abstract

Nine Peruvian isolates of *Vibrio parahaemolyticus* were characterized through sequencing, revealing the presence of simple sequence repeat, Pir toxin-like genes, and genes associated with antibiotic resistance, toxic components, and transposable elements. These findings expand our understanding of the genetic diversity, disease resistance, and virulence in cultivated shrimp populations in Peru.

## ANNOUNCEMENT

Acute hepatopancreatic necrosis disease (AHPND) in *Penaeus vannamei* shrimp is specifically caused by strains of *Vibrio parahaemolyticus* (family Vibrionaceae) carrying a virulent plasmid pVA1, encoding the PirAvp and PirBvp toxins (1–3). In Latin America, pathogenic strains have been identified, but high mortalities as observed in Asia have not been reported. In this region, a chronic disease with unique tolerance to AHPND has been described in the Latin American shrimp population (4).

In this study, nine samples were collected, each containing five juvenile shrimp specimens. These samples were obtained from four epidemiological zones in Tumbes, Peru, between April, May, and August 2022. The epidemiological zones included North Zone 1 (samples Tumbes-M02, Tumbes-M04, and Tumbes-M05), South Zone 1 (Tumbes-M03), South Zone 2 (Tumbes-M06), and Central Zone (Tumbes-M07, Tumbes-M10, Tumbes-M11, and Tumbes-M12). Hepatopancreas tissue was collected and enriched in Trypticase soy broth medium with 2.5% NaCl, followed by a 16-hour incubation at 30°C. Subsequently, the bacterial culture was plated on CHROMagar plates. Mauve-colored colonies were selected and then inoculated onto thiosulfate citrate bile sucrose agar (TCBS) plates, from which green-colored colonies were chosen for further analysis.

DNA from the selected colonies was extracted through thermal shock, involving heating at 100°C for 10 minutes, followed by cooling at and −20°C for 2 minutes. Molecular detection of PirAB genes was carried out using PCR PirAB Duplex (4). Subsequently, nine confirmed colonies were selected for sequencing. Total DNA was isolated from bacterial cultures using the NucleoSpin tissue DNA kit (Macherey-Nagel, Germany) and treated with RNase. The isolated DNA was quantified using the Qubit4 fluorometer. The library was prepared using the Rapid Barcoding kit (SQK-RBK004) following the manufacturer’s instructions and loaded into an R9.4.1 flow cell. Sequencing was conducted on a MinION Mk1C device (Oxford Nanopore Technologies, UK) for a duration of 25 hours (1.58 million reads; 9 gigabases of passed basses; N50: 11.25 kb; average QScore: 11). Basecalling was performed using Guppy v5.0.12 (Fast basecalling).

The obtained sequences were processed using default parameters unless otherwise specified. In the Galaxy platform (5), the sequences were concatenated using the “Concatenate data sets” program (Galaxy Version 0.1.1) (6). *De novo* assemblies were performed using the Flye program (Galaxy Version 2.9.1+galaxy0) (7), and the taxonomic identity of the isolates was determined using Kraken2 (Galaxy Version 2.1.1+galaxy1) with the k2_standard_20210517 database (8). The results were visualized with Krona (Galaxy Version 2.6.1.1) (9), and annotations were conducted using the NCBI Prokaryotic Genomic Annotation Pipeline (PGAP) v.4.8 (10) and RASTtk (11) for functional analysis with the following options: “RASTtk: Automatically fix errors,” “Fix frameshifts,” and “Backfill gaps on YES.” During the analysis, genes associated with antibiotic resistance, toxic components, and transposable elements such as phage fragments and integrons were identified. Furthermore, the presence of SSR (TTGTTTTTC) with five and six repeats was found manually in plasmids carrying PirAB genes (1) (Table 1).

**TABLE 1:** Genomic characterization of *Vibrio parahaemolyticus* isolates from the Tumbes region^*a*^

Isolate	Assemblies					Subsystem feature counts	
	N° contigs	Length (bp)	Cov.	Circ.	%GC	Resistance to antibiotics and toxic compound	Transposable elements
**Tumbes-M02** (**JAUIQE000000000**)	contig_2	3,329,736	262	Y	45	Copper homeostasis (18) Cobalt-zinc-cadmium resistance (8) Resistance to fluoroquinolones (2) Copper homeostasis: copper tolerance (9) Resistance to chromium compounds (2) Multidrug resistance efflux pumps (19)	Phage replication (3) Prophage-encoded Rst operon (2) Phage DNA synthesis (3) Integrons (2)
	contig_1	1,906,514	201	Y	45		
	contig_7^*c*^	54,879	766	N	43		
	contig_8	51,645	825	N	44		
	contig_3	23,633	575	N	52		
	contig_6	4,672	830	Y	41		
	contig_5	4,083	134	N	50		
**Tumbes-M03** (**JAUIQF000000000**)	contig_1	3,366,142	262	Y	45	Copper homeostasis (17) Cobalt-zinc-cadmium resistance (7) Resistance to fluoroquinolones (2) Copper homeostasis: copper tolerance (10) Resistance to chromium compounds (1) Multidrug resistance efflux pumps (16)	Phage replication (7) Phage DNA synthesis (3) Integrons (2)
	contig_3	1,958,396	204	Y	45		
	contig_5^*b*^	73,251	681	Y	45		
	contig_4	57,864	749	Y	47		
	contig_2	21,938	638	N	52		
**Tumbes-M04** (**JAUIQG000000000**)	contig_2	3,363,672	72	Y	45	Copper homeostasis (15) Cobalt-zinc-cadmium resistance (7) Resistance to fluoroquinolones (2) Copper homeostasis: copper tolerance (9) Resistance to chromium compounds (1) Multidrug resistance efflux pumps (12)	Phage DNA synthesis (3) Integrons (2)
	contig_5	1,958,928	58	Y	45		
	contig_4^*b*^	73,294	95	Y	46		
	contig_1	57,904	81	Y	46		
	contig_3	21,947	432	N	52		
**Tumbes-M05** (**JAUIQH000000000**)	contig_1	3,366,948	113	Y	45	Copper homeostasis (15) Cobalt-zinc-cadmium resistance (8) Resistance to fluoroquinolones (2) Copper homeostasis: copper tolerance (9) Resistance to chromium compounds (1) Multidrug resistance efflux pumps (14)	Phage replication (6) Phage DNA synthesis (3) Integrons (2)
	contig_3	1,958,861	85	Y	45		
	contig_4^*b*^	73,299	210	Y	46		
	contig_2	57,869	229	Y	46		
	contig_5	21,930	506	N	52		
**Tumbes-M06** (**JAUIQI000000000**)	contig_1	3,366,917	119	Y	45	Copper homeostasis (15) Cobalt-zinc-cadmium resistance (8) Resistance to fluoroquinolones (2) Copper homeostasis: copper tolerance (9) Resistance to chromium compounds (1) Multidrug resistance efflux pumps (14)	Phage replication (6) Phage DNA synthesis (3) Integrons (2)
	contig_3	1,957,184	95	Y	45		
	contig_4^*b*^	73,304	243	Y	46		
	contig_5	57863	275	Y	46		
	contig_2	21937	537	N	52		
**Tumbes-M07** (**JAUIQJ000000000**)	contig_1	3367212	74	Y	45	Copper homeostasis (15) Cobalt-zinc-cadmium resistance (7) Resistance to fluoroquinolones (2) Copper homeostasis: copper tolerance (9) Resistance to chromium compounds (1) Multidrug resistance efflux pumps (15)	Phage replication (5) Phage DNA synthesis (3) Integrons (2)
	contig_3	1957361	59	Y	45		
	contig_5^*b*^	74470	161	N	46		
	contig_4	35928	168	N	43		
**Tumbes-M10** (**JAUIQK000000000**)	contig_1	3366564	181	Y	45	Copper homeostasis (17) Cobalt-zinc-cadmium resistance (7) Resistance to fluoroquinolones (2) Copper homeostasis: copper tolerance (9) Resistance to chromium compounds (1) Multidrug resistance efflux pumps (17)	Phage replication (5) Phage DNA synthesis (3) Integrons (2)
	contig_3	1958634	141	Y	45		
	contig_4^*b*^	73267	375	Y	46		
	contig_2	57878	332	Y	46		
	contig_5	21927	539	N	52		
**Tumbes-M11** (**JAUIQL000000000**)	contig_1	3391395	189	Y	45	Copper homeostasis (17) Cobalt-zinc-cadmium resistance (6) Resistance to fluoroquinolones (2) Fosfomycin resistance (1) Copper homeostasis: copper tolerance (9) Resistance to chromium compounds (1) Multidrug resistance efflux pumps (17)	Phage replication (4) Phage packaging machinery (2) Phage DNA synthesis (3) Phage tail proteins (11) Phage tail proteins 2 (11) Prophage-encoded Rst operon (2) Phage capsid proteins (1) Phage tail fiber proteins (2) Integrons (2)
	contig_2	1907405	152	Y	45		
	contig_7	124986	280	Y	44		
	contig_6^*b*^	73218	335	Y	46		
	contig_20	42402	272	Y	48		
	contig_18	34908	372	Y	46		
	contig_17	21189	795	N	53		
	contig_16	19712	504	N	42		
	contig_11	13187	575	N	40		
	contig_8	13063	700	N	46		
	contig_22	11506	564	Y	41		
	contig_14	9335	588	Y	41		
	contig_10	7614	265	N	41		
	contig_21	7127	586	Y	42		
	contig_15	5836	317	N	39		
	contig_3	2494	54	N	45		
	contig_9	1747	67	N	42		
**Tumbes-M12** (**JAUIQM000000000**)	contig_15	3391992	101	Y	45	Copper homeostasis (17) Cobalt-zinc-cadmium resistance (7) Resistance to fluoroquinolones (2) Fosfomycin resistance (1) Copper homeostasis: copper tolerance (10) Resistance to chromium compounds (1) Multidrug resistance efflux pumps (12)	Phage tail proteins (6) Phage replication (3) Phage packaging machinery (2) Prophage-encoded Rst operon (2) Phage tail proteins 2 (6) Phage tail fiber proteins (2) Phage DNA synthesis (3) Integrons (1)
	contig_21	1907735	84	Y	45		
	contig_5	125091	133	Y	44		
	contig_6^*b*^	46703	314	N	43		
	contig_22	42405	432	Y	48		
	contig_3	29192	273	N	42		
	contig_18	21103	421	N	53		
	contig_17	20017	34	Y	40		
	contig_1	17447	882	Y	46		
	contig_12	13510	314	N	40		
	contig_10	13068	599	N	46		
	contig_20	11502	510	Y	41		
	contig_4	10880	347	N	41		
	contig_16	9330	585	Y	41		
	contig_2	4240	129	N	44		
	contig_19	3716	90	N	55		
	contig_14	3593	819	Y	42		
	contig_7	2372	89	N	42		

^*a*^ This table presents a comprehensive genomic analysis of *Vibrio parahaemolyticus* isolates obtained from the Tumbes region (Peru). Essential details are provided, including isolate names, contig names, contig lengths in base pairs (bp), coverage (cov.), circularity (circ.), GC content (%GC), resistance to antibiotics and toxic compounds, and transposable elements.

^*b*^ The presence of the PIrA gene and the simple sequence repeat (SSR) repetitive sequence (TTGTTTTTC) five times is indicated.

^*c*^ Additionally, the presence of the PIrAB gene and the SSR repetitive sequence six times is also indicated.

This study contributes to the understanding of the pathogenicity and evolution of these bacteria in the context of diseases in farmed shrimps. These findings may have significant implications for the development of strategies for disease control and prevention in aquaculture.

## Data Availability

The Nanopore raw reads for this sequencing project (PRJNA980916) are available under the following accession numbers: SRR24899490, SRR24899239, SRR24899244, SRR24898281, SRR24899242, SRR24899243, SRR24898079, SRR24899231, SRR24902115. The assembled contigs can be accessed using the following accession numbers: JAUIQE000000000, JAUIQF000000000, JAUIQG000000000, JAUIQH000000000, JAUIQI000000000, JAUIQJ000000000, JAUIQK000000000, JAUIQL000000000, JAUIQM000000000.
